# ME^3^CA: A Cognitive Assistant for Physical Exercises that Monitors Emotions and the Environment

**DOI:** 10.3390/s20030852

**Published:** 2020-02-05

**Authors:** Jaime A. Rincon, Angelo Costa, Paulo Novais, Vicente Julian, Carlos Carrascosa

**Affiliations:** 1 Department of Computer Systems and Computation, Universitat Politècnica de València, 46022 Valencia, Spain; jrincon@dsic.upv.es (J.A.R.); vinglada@dsic.upv.es (V.J.); carrasco@dsic.upv.es (C.C.); 2 ALGORITMI Center, University of Minho, 4700 Braga, Portugal; pjon@di.uminho.pt

**Keywords:** cognitive assistants, affective computing, elderly

## Abstract

Recent studies show that the elderly population has increased considerably in European society in recent years. This fact has led the European Union and many countries to propose new policies for caring services directed to this group. The current trend is to promote the care of the elderly in their own homes, thus avoiding inverting resources on residences. With this in mind, there are now new solutions in this direction, which try to make use of the continuous advances in computer science. This paper tries to advance in this area by proposing the use of a personal assistant to help older people at home while carrying out their daily activities. The proposed personal assistant is called ME^3^CA, and can be described as a cognitive assistant that offers users a personalised exercise plan for their rehabilitation. The system consists of a sensorisation platform along with decision-making algorithms paired with emotion detection models. ME^3^CA detects the users’ emotions, which are used in the decision-making process allowing for more precise suggestions and an accurate (and unbiased) knowledge about the users’ opinion towards each exercise.

## 1. Introduction

The current trend in our society is for elderly people (over 65 years) to surpass the number of births [1]. This assumes that the worldwide population is ageing rapidly. With this progression, soon the population will find difficulties to receive social assistance for the elderly. This would be mainly due to the lack of funds for the decrease in the number of people who are available to contribute monetarily to the society. Another problem will be the lack of social/medical workers, as commented in [2]. Families will have many complications in caring for the elderly themselves or through caregiving services [2].

Social services are currently unable to provide care for all elderly people who need them and this issue will be even more critical in the future [3]. Adapting these services will have a high cost due to the demand for infrastructures and specialised personnel [4]. By 2060, 44.4 million people of over 65 years will need assistance on their activities of daily living (ADLs) [5].

Today, the growing number of older people with cognitive problems, such as Alzheimer’s, aggravates this problem. This aspect is commented in the study presented in [6]. Caring for people with these cognitive problems requires special training and that is difficult to find in today’s caregivers of older people in the home setting [4]. Besides, the high level of care these elderly people need makes it impossible for caregivers to care for more than one person [5]. In this case, we refer to correct medication intake and adequate medical control.

A possible solution may be to adapt to the elder’s home by using technological devices to increase the duration of stay of these people in their own homes more comfortably and safely. Also, keeping older people in their homes has great benefits [7]. On the one hand, they know this familiar environment well and the displacements are shorter, which is very important because of the high risk of falls. Besides, their family and neighbours can visit them more frequently. On the other hand, being at home can have negative consequences such as the lack of exercise doing repetitive and unchallenging tasks. Typically these tasks are often associated with cognitive problems like dementia [8]. To overcome this problem, a solution passes through the promotion of exercises and activities that involve the elderly in the practice of physical and cognitive exercise.

The exercises to be recommended should be meaningful (or helpful) for the elderly and should have a positive impact on their day-to-day life. Moreover, the performed exercises should be adequately controlled, meaning the monitoring of the level of the elderly health state (through sensor devices) and their environment (for external exercises/activities).

To overcome all of these issues, this paper presents ME^3^CA, a personal assistant whose goal is to plan and recommend activities to older people who are trying to improve their physical activity. To achieve this, the assistant makes use of the quality of the environment and the emotional state of the user to improve the decision-making process. This implies the usage of personalising methods in the assistant’s suggestions, as well as creating an emotional connection with the users’ thanks to the human-like emotions expression by the assistant. The social goal is to maintain the elderly people in the comfort of their own homes, thus avoiding moving them to dedicated facilities, and to closely monitor their health condition forecasting possible critical problems and acting proactively. This project contributes to the state of art by introducing emotional monitoring in relation to physical activities. Humans tend to be more kind when responding verbally or in writing to questionnaires being quite inaccurate. Through the use of noninvasive sensors (e.g., not using cameras) ME^3^CA attains unequivocal information about the emotions of the sensor-wearing users.

This paper is structured in the following manner. Section 2 analyses previous works in the related area. Section 3 shows the proposed system detailing both the hardware and software components. Section 4 describes the validation of the proposed models. Section 5 shows an evaluation study of the proposed system. Finally, Section 6 presents some conclusions and future work.

## 2. Related Work

Although physical exercises are regarded as one of the most effective way to maintain the health condition of the elderly (both cognitive and physical), technology is only very recently catching up with solutions for intelligently suggest and monitor people performing exercises. Furthermore, exercise suggestion was just a guideline-based method, although currently these suggestion systems are being upgraded to encompass new features like sensor systems and artificial intelligence procedures, following the path of well known decision support systems applied in healthcare [9]. In this section, we present the most relevant projects and platforms in relation to ME^3^CA.

The platform Physical Activity Monitoring for Aging People [10] uses a mixture of the environmental and bodily sensors to ascertain if the elderly are performing correctly the exercises. The objective of this project is to monitor the elderly so that a caregiver is not actively needed when they are preforming these exercises. The platform also provides information to the elderly about the exercise schedule and how to perform those exercises. To assert if the elder is performing correctly these exercises, a comparative between correct exercises pattern and the exercise pattern is done (having some error tolerance). This is done in the following way; during the platform set-up, the elder performs the exercises with the help of the caregiver and these movement are registered in the system; in the following exercises performances the movements of their gyroscopes are compared to the basal system and warnings are displayed if the values are out of the threshold. While the platform works with personalised profiles, they do not evolve and are the same since the platform set-up.

In the same line is the PersonAAL project [11] that monitors the elder’s behaviour and displays health-related suggestions searching for any decay of motor abilities. It uses environmental sensors (an AAL-enabled house) and body sensors to detect any values that are above of the threshold. The objective is to have web applications so that users receive personalised and context-dependent assistance directly in their homes, and to allow the caregivers to monitor the elders in an intuitive manner. Currently, according to recent publications, the platform has a body sensor network and environment sensors and a functional web, but not effective personalisation or any learning methods.

Using visual monitoring there is the platform HemoKinect [12] that uses a Kinect camera to monitor people with haemophilia. They capture the colour (RGB) image and depth map to identify human joints. The objective is to trace the user’s movement and calculate the centre-of-mass, avoiding potential falls and injuries. Currently, their calculations are the direct outcome of angles and distance to the floor. The platform still lacks a visual interface and reporting methods for the caregivers. Furthermore, no personalising is performed to accommodate the different user’s profile.

The Salvio project [13] consists of a web platform that aggregates external services (HEART [14], Text4Heart [15], TEXT ME [16], and REMOTE-CR [17]) with the objective of providing a personalised experience to each user. Each user goes through a questionnaire that identifies their health problems and redirects them to the appropriate above mentioned services. The user’s can observe their improvement evolution and activate/deactivate services that they see fit. For instance, REMOTE-CR [17] is composed by a wearable sensor, a mobile phone and a web page. It provides the users with coaching from clinical exercise specialists directed towards exercising in their homes. The main difference it has to commercial applications is that it has behavioural guidelines that are evidence and theory-based. The sensor is a Zephyr BioModule (https://www.zephyranywhere.com/system/components) that has the following features; heart rate, heart rate variability, breathing rate, posture, activity level and approximate core temperature. With this data, the REMOTE-CR gives real-time advice to improve the exercise performance as well as build an historic that the user’s can access through the web portal.

Using a robot for sensing and interfacing is the PHAROS project [18]. It uses a Pepper Robot to teach and evaluate physical exercises providing this information to the caregivers, using its friendly appearance to engage with the elderly. It has a high-level of personalisation and has a recommending system is able to learn from the user’s performance or if prompted by the caregivers. The movements of the elder are captured by the robot’s cameras, and using deep leaning methods, a projection of the body position is performed and verified if it is within the expected threshold. The robot is used as a companion, and its exercise suggestion triggered in specific times where it is expected that the elderly are more available to perform activities. Furthermore, the caregivers are gifted with an web interface were they can see reports of the exercise performance and critical issues are displayed, and can adjust the suggestions or change the thresholds. It does not verify directly the elder’s vital signs, possibly missing important data about their health condition.

Finally, there are several commercial applications, like Runtastic (https://www.runtastic.com), Google Fit (https://www.google.com/fit/), Noom (https://www.noom.com), Fitbit (https://www.fitbit.com) and Health Mate (https://www.withings.com), that promise personalised (either automatically or via a personal trainer) exercises. Unfortunately, they are not adapted to elderly people nor have explicitly formal medical approval nor any evidence and theory-based approach towards the recommendation.

ME^3^CA is designed to overcome most of the presented projects issues by being a personalised exercise recommendation platform that monitors the users via low-cost body sensors and environmental sensors while tracking the emotional state of the users for profile enhancement. Furthermore, ME^3^CA detects the users’ emotions that are used in the decision-making process allowing for more precise suggestions and an accurate (and unbiased) knowledge about the users’ opinion towards each exercise.

These features are explained with detail in the following sections.

## 3. System Description

**Figure 1:**
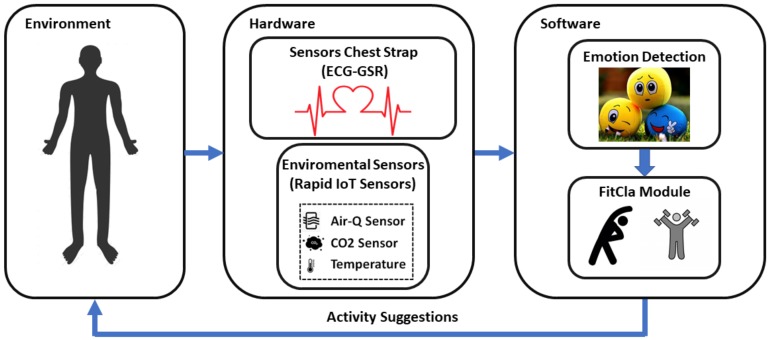
ME^3^CA’s components.

This section describes the different parts that make up our ME^3^CA system, see Figure 1. ME^3^CA has been divided into the user, a hardware part and a software part:

- Hardware: This group is composed of sensors for acquiring the different physiological signals, and a set of environmental sensors. These sensors can be used to perceive the evolution of the person to the exercise sequence. In fact, these sensors are grouped in two different artefacts: the Sensors Chest Strap, formed by a set of sensors that will go in the user chest, and they even could be linked to a slim-fit exercise t-shirt; and the Environmental Sensors formed by a set of sensors that can collect environmental information. Regarding the Sensors Chest Strap, it allows the acquisition of physiological signals such as electrocardiography, photoplethysmography and the calculation of the cardiac pulse. This information will, then, be used to perform a recognition of the individual’s emotional state [19,20]. The Environmental Sensors allows the system to determine if the environmental signs are optimal for performing the exercises. They measure conditions such as temperature levels, humidity, CO2 and air quality among others, as high levels of these environmental factors can cause harmful effects when exercising [21,22,23].
- Software: This group is formed by an external service in charge of processing the different signals and adapting a sequence of personalised exercises for each individual. To dynamically adapt the exercises, the system requires a series of data provided by the sensors. This software is divided into two modules: *Empathy Module*, according to the sensor information, calculates the current user’s emotion, and the *FitCLA*, which calculates the proper exercises’ sequence or adapts the current one according not only to the user profile, but also his current physical and emotional stress.

The rest of the section is going to detail the hardware and software parts of the system.

### 3.1. Hardware Description

This section presents the description of the hardware, which, as commented above, has been divided into two subsystems. The first is composed of a chest belt, which incorporates a series of sensors. These allow us to acquire the signals of ECG (Electro Cardiography) and GSR (Galvanic Skin Response). The second is composed of a bracelet, which incorporates sensors of temperature, humidity, motion detector, fall detector, air quality and CO2 levels.

The data acquired by these two systems are sent to a web service to be analysed in depth, using AI techniques such as Deep-Learning [24] or Neural Networks [25]. The network topology used to classify the activities is described in detail in the Section 4. This capacity to classify the activities allows to make a pursuit of the activities, allowing to modify them in real-time, obtaining therefore a penalisation of the activities.

**Figure 2:**
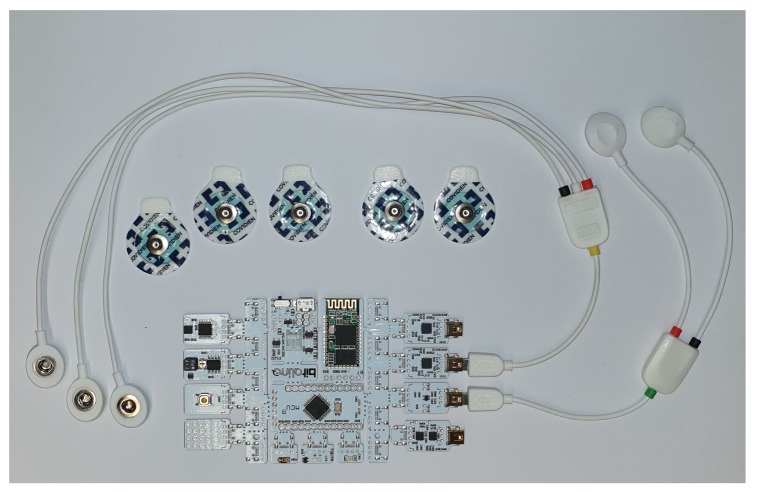
Bitalino development system.

To acquire the physiological signals necessary to classify emotions, the Bitalino (https://bitalino.com/en/) development system was used. This system incorporates different biosignal acquisition systems, of which the ECG and EDA modules were used. The system can be seen in Figure 2. The Bitalino has a bluetooth interface that is used to send the acquired data to the smartphone. Through an application it is able to send it to the proper web service.

**Figure 3:**
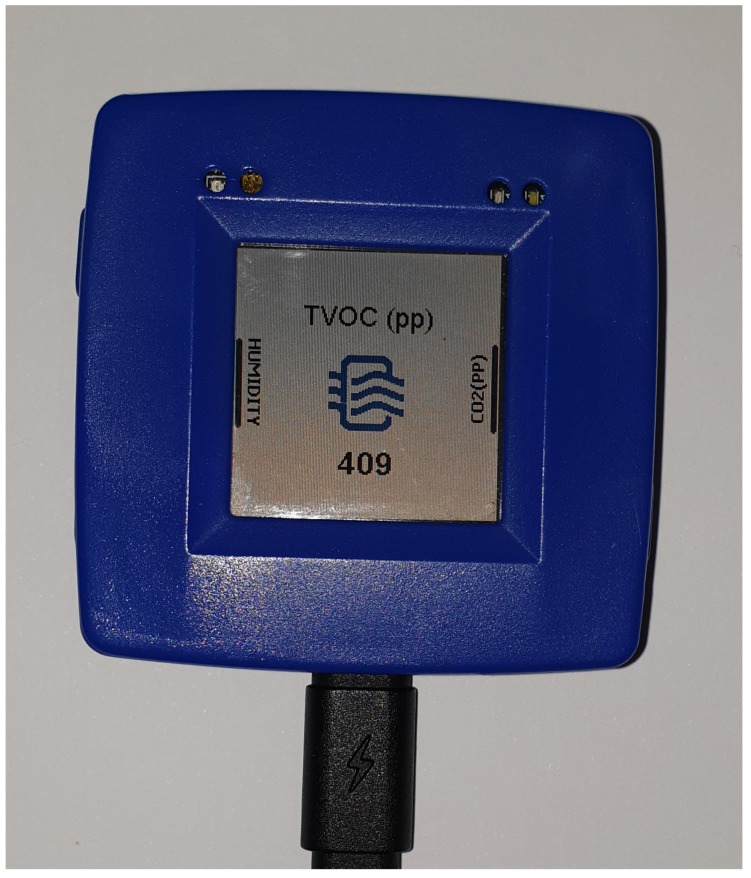
Rapid IoT development system.

On the other hand, the bracelet has been developed using the Rapid IoT development system (Figure 3) of the NXP (https://www.nxp.com/support/developer-resources/rapid-prototyping/nxp-rapid-iot-prototyping-kit:IOT-PROTOTYPING) company.

A new app allowing to connect the bracelet to an smartphone has been created. This app allows acquisition and observation of the signals in our phone. At the same time, it is possible to configure the app, so that it sends the information acquired to our web service. In this way, the acquired information can be stored in a database for later analysis. At the same time, our web service uses these data, to enhance the respective recommendations of activities.

Once the signals have been acquired and preprocessed, they are sent to the web-service. This web-service uses different AI tools to analyse the signals and try to detect emotional states; stress; or heart problems such as arrhythmia, tachycardia or bradycardia.

### 3.2. Software Description

This section describes the various software tools used in this article. The software tools used have been divided into two subsections. The first one describes the classification of emotional states using Deep-learning, whereas the second one describes the FitCLA system.

#### FitCLA Module

The hardware is complemented by the FitCLA module, used for visualisation and exercises suggestion. Currently, the FitCLA is still undergoing through the development phase and concurrently being tested by elderly people to assert if the suggestions/interface is appropriate. Therefore, we are in still in the process of validation, thus stable results are still unavailable.

FitCLA is the outcome of continuous developments presented in [18,26,27,28]. It is a web-service that can be easily integrated in platforms, using sensor systems to generate knowledge about the users profile and provide suggestions according to them. Thus, FitCLA is a cognitive assistant that uses profiles to give suggestions, learning from the positive/negative responses and sensor systems, and adapting to the user’s physical impairments (e.g., memory loss and assisted mobility).

**Figure 4:**
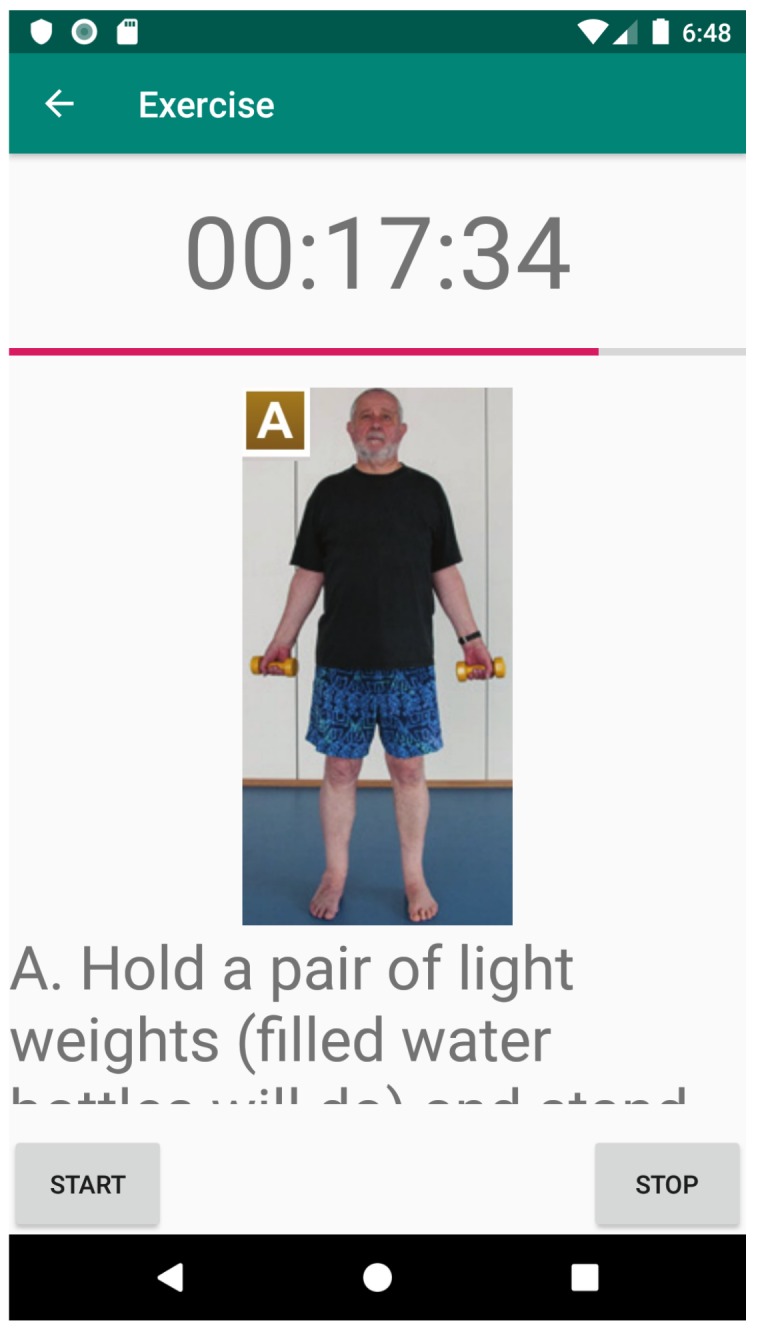
Care-receiver exercise information (part 1).

**Figure 5:**
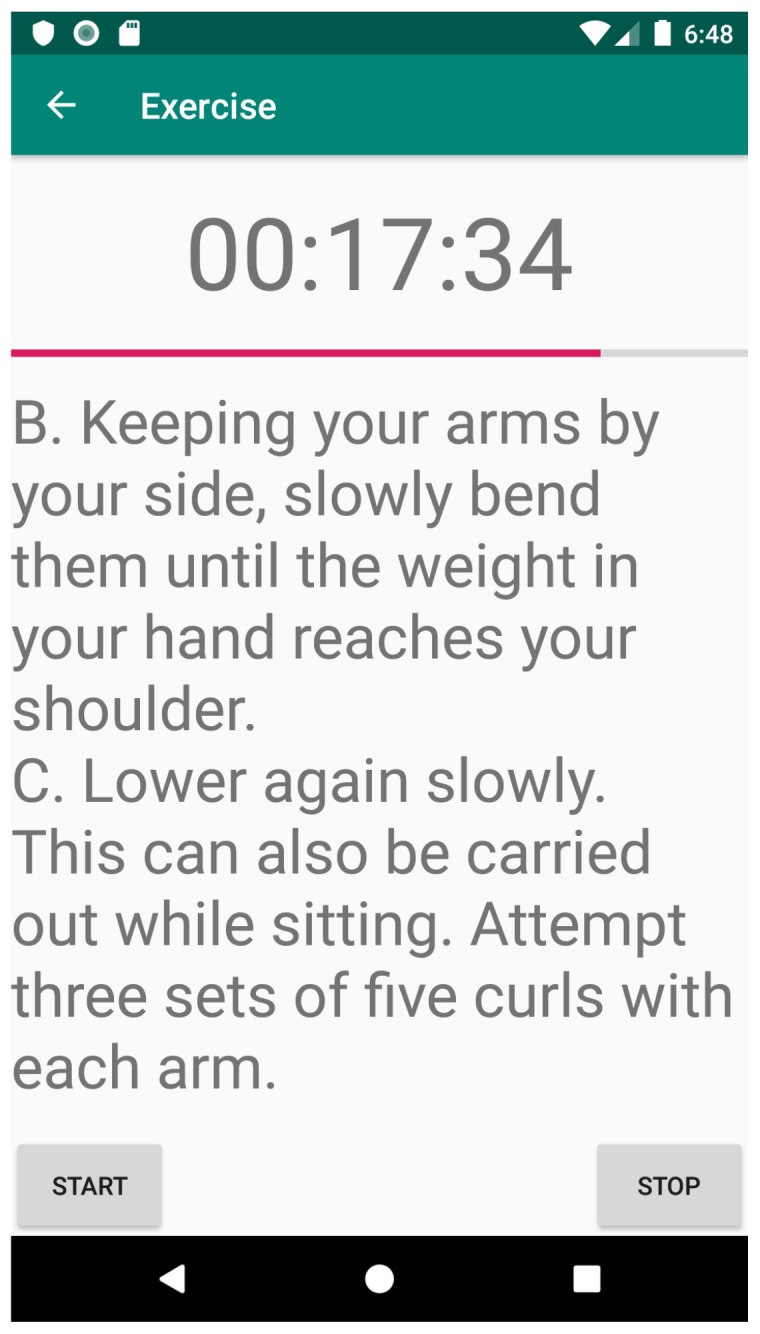
Care-receiver exercise information (part 2).

Apart from a scheduler (to add new exercises to the user’s daily life), based on the iGenda [27], FitCLA is composed by two more features: visual interfaces and learning models. Currently, these two methods are under a through validation process as both can greatly impact the user’s adoption rate and operational procedures. Preliminary visual interfaces can be observed in Figure 4 and Figure 5. These two interfaces show how users (in this case, the care-receivers) can visualise an exercise and activate the timer and guides that help them during the exercise performance. This helps the emotion recognition module and exercise identification, as the users signal when they have started to perform the exercise, which serves to coordinate the sensors log. The caregivers have also a dedicated web portal where they can manage the care-receivers information, exercise suggestion and overall configuration. Any changes in this portal (e.g., scheduling times) are reflected immediately on the mobile application. Furthermore, any alarms are directly shown to the caregiver. In ME^3^CA, the information is displayed both in the bracelet screen as well as on the mobile phone. A short text is showed along with a start/stop button so the user is able to signal when the exercise has started and stopped accordingly.

FitCLA uses the information from the sensor systems in the following way; to access if the care-receivers are performing correctly the exercises, and to verify the emotional status of the care-receivers, thus their opinion towards the exercise proposed. This information is then used in learning models which in turn are used in the filter process of the recommendation.

To adjust the suggestions to the user’s preferences and needs, FitCLA uses learning models. It is composed of two learning processes. They are still under scrutiny due to the low number of samples, and thus, currently, underperforming in relation of what are acceptable values. To predict the user’s mood we have chosen the Random Forest (RF) method. First, the data is preprocessed and the emotion values changed to binary values. We have divided the emotions into two groups: positive and negative. Then the rest of the categorical data is transformed with One Hot Encoding for simplicity. Finally, the RF is created. This procedure is not without its shortcomings like the significant loss of information (emotion). This approach was chosen over others as it was the one that produced the best results. Currently the accuracy value is 67%, a sub-par result, but without more samples no improvement can be achieved. To predict the exercises it is used a Linear SVC model. This was chosen over other methods due to its simplicity and the small amount of classes (amount of exercises) that are available. In this model, the emotion field is dropped and only values related to the physical condition (arms/legs problems, etc.) and age/gender are considered. This was done to reduce the possible noise and to have a more direct result. It was used a linear kernel with a gamma value of 25. Again, with the small amount of samples the accuracy values suffer greatly.

The learning methods are underperforming, and validation is still not possible as we are building a completer dataset to test other (more robust) methods to achieve better results.

The cold start of the system is performed by data input by the caregivers. At an initial phase, FitCLA receives a set or user profiles that are composed initially by registered caregivers that assert the level of performance for each user (0 to 100) on each exercise the system has (FitCLA uses the NHS recommended list exercises [29]). These profiles can be changed at any time by the caregivers. Each exercise has a starting value of 50 to emotion to each user, being this value changed according to the user emotional response when performing the exercise.

FitCLA receives information from the sensor platform and the *Emotion Recognition Module* when the exercise is performed and with this data it recalculates the scores to each exercise. For the exercises (overall performance), FitCLA uses thresholds and a limited variance values for activation. Finally, when the threshold is reached an alarm is sent to the caregivers.

## 4. Emotion Recognition Validation

This subsection describes the elements that make up the software which allows the classification of emotional states using different biosignals and the recommendation of activities through these emotional and environmental data. In order to make this recommendation, it is necessary to classify the emotions presented by the users of the system. This classification was done using Deep-Learning as a machine learning tool. For this purpose, the different biosignals (ECG, GSR and PPG) are used as input data, which were acquired by sensors located on the chest and bracelet.

These signals were preprocessed to eliminate noises such as 50 Hz electrical noise and possible noise produced by involuntary movements generated by the user. To determine which frequencies affect our signal, we used the Fourier Fast Transform, which allowed us to visualise the harmonics that affect our signal. To eliminate these harmonics that distort the signal, we used different configurations of digital IIR filters (band pass and band-stop).

In order to carry out the training phase, the AMIGOS database [30] was used. This database has a collection of signals from Electro Encephalography (EEG), Galvanic Skin Response (GSR) and Electro Cardiography (ECG).

These signals (ECG and GSR) were obtained through users, who were submitted to visual and auditory stimuli. At the same time, users responded to a self-assessment form to extract information related to levels of excitement, valence, mastery, taste, familiarity and basic emotions (Neutral, Happiness, Sadness, Surprise, Fear, Anger and Disgust). Only the ECG and GSR signals were taken into account for the training phase.

It was decided to use these two signals because they are easy to acquire with a low-level of intrusion to the user using the device, comparing to EEG signals, which require more specialised hardware and a high-level of intrusion (it is necessary to have a professional that place the 32 or 64 electrodes on the patient’s head). Taking into account this signal restriction, a new database was built in which the ECG and GSR signals of all users of the experiments were stored. To extract the main features to this new database, the equations presented by Picard [31] were used.

Picard defines equations to extract six characteristics using statistical methods. Based on these equations, we extracted these six characteristics from our ECG and GRS signals. These characteristics are then used as input for the emotion classification algorithm, which uses Deep-learning as a tool to perform this classification. The network built for this purpose was a one-dimensional convolutional network (CNN-1D). Another advantage of using these equations is the reduction in signal length, as the signals used for training are 8064 in length.

**Figure 6:**
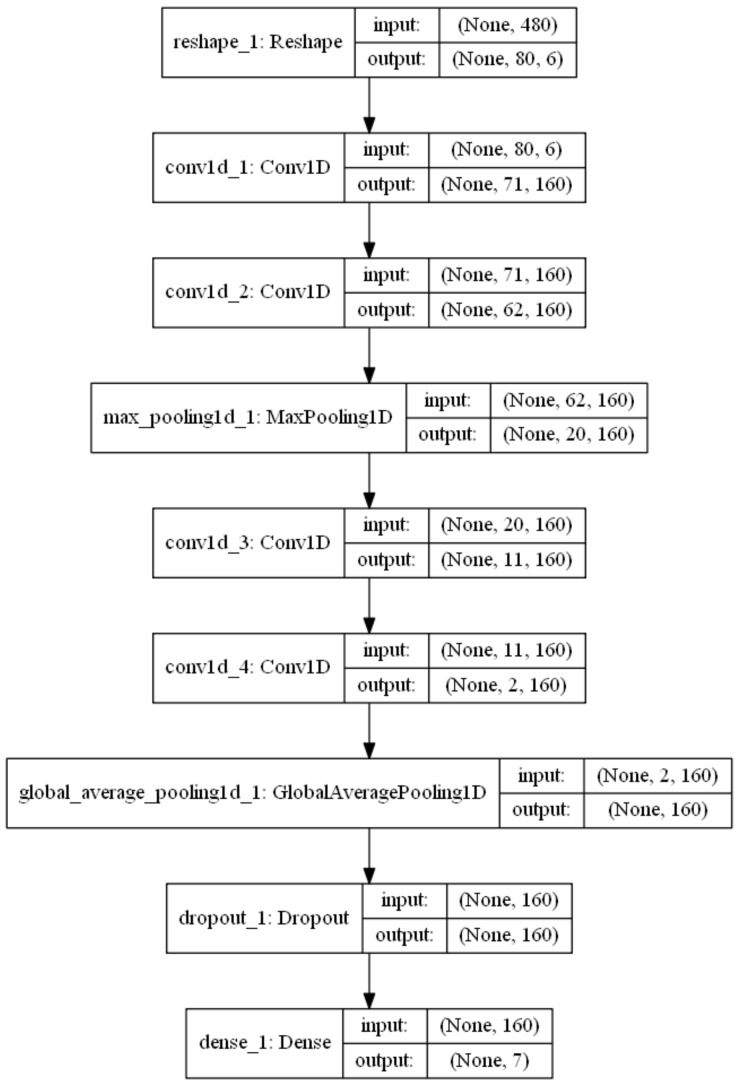
Structure of the convolutional neural network.

A series of experiments were carried out using different hyper-parameters till finding the ones in Table 1. The model with the best results was the one shown in Figure 6, and corresponds to the one with the before commented hyperparameters.

The basic emotions noted by the users were used as the output elements of the Deep Learning process. To be processed as output, those basic emotions were categorised in binary.

The extraction of these data allowed us to build a new set of data, with which we have trained the network. From this restructuring of our data, we have created a dataset with 784,000,000 elements. This new dataset is different from the one used in the FitCla, that’s why the number of data to perform the training is higher. These dataset were divided into three subsets: training, test and validation, with a proportion of *Training = 80%, Test = 10% and Validation = 10%*. The distribution of the dataset data was as follows; *Training = 627,200,000, Test = 78,400,000 and Validation = 78,400,000*.

**Figure 7:**
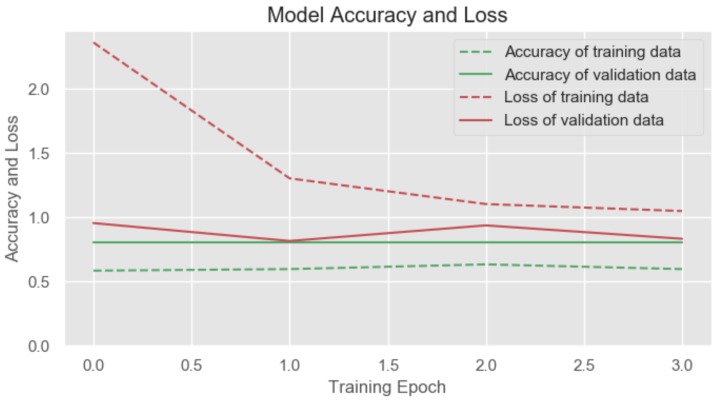
Model accuracy and loss.

From the training process of our network, we extracted the parameters of prediction and loss. These were obtained from the training and validation data and are shown in Figure 7. The red dotted line shows us the loss in the data in the training phase, whereas the continuous line of the same colour indicates the loss in the validation data. It can be observed that our loss in the training phase is greater than in the validation phase. This allows us to determine that there is no overtraining. As the loss in the validation phase is lower, we can determine that our model classifies correctly and since there is no overtraining, we conclude that the model has been adapted to the training data.

## 5. System Evaluation

This work has been tested by caregivers and patients of a daycare centre in the north of Portugal (Centro Social Irmandade de S. Torcato). Different experiments were performed and then analysed. The objective was to analyse the performance of ME^3^CA and the user acceptance of it. Concurrently, we gathered data for our dataset taking advantage of enriched labels.

The experiments were performed with the following methodology in a categorical manner.

- A small group of care-receivers has been monitored by the system for two weeks.
- Three activities have been proposed to each care-receiver during each day in different periods of the day.
- The care-receivers have filled out a simple questionnaire about how they felt before and after doing the proposed activities.
- All activities have been supervised by specialised caregivers.
- Caregivers have also done a questionnaire on the appropriateness of the recommended activities in each case.
- The results obtained have been analysed by comparing the opinions of older people and caregivers.

Both the elderly and caregiver questionnaires have been designed to be as simple as possible (especially in the case of the elderly) and using Likert Scales. This has been done to reduce the entropy of the answers and to linearise the values obtained. The questions designed for the two questionnaires are shown in the Table 2 for the elderly and in the Table 3 for the caregivers.

The tests were performed by 15 persons between the ages of 65 and 70 and five caregivers. As commented, after each activity is completed, the care-receivers and the caregiver filled out the questionnaires. At the end of the experiment, an analysis was made of the suggestions offered by the system and the opinions of the participants through the questionnaires.

**Figure 8:**
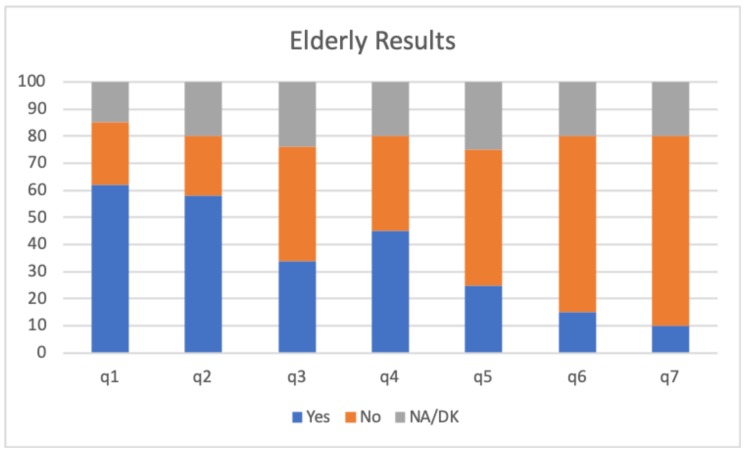
Results obtained from the elderly questionnaire.

**Figure 9:**
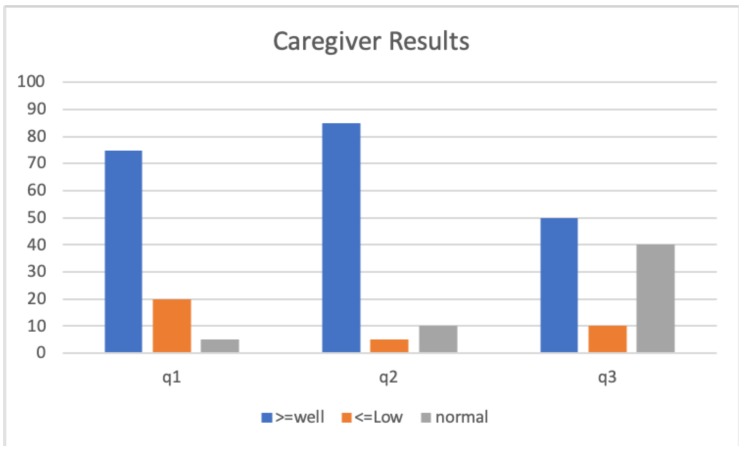
Results obtained from the caregiver questionnaire.

The results obtained from this study are presented in Figure 8 and Figure 9. Specifically, Figure 8 shows the results taking into account the average of the responses of the elderly to the questionnaire. As it can be seen, Q1, Q2 and Q3 are related with the adequacy of the activity with respect to the care-receiver, which showed a fairly acceptable result. In general, the recommended activities have been to the liking of people and they have felt good after performing the activity. Regarding Q4–Q7 (related to the emotional state after the activity), it is observed that a significant percentage (45%) have felt excited after performing the activity recommended by the system and that the percentages of the rest of the answers are quite low, which is positive.

Concerning the results obtained in Figure 9, we can observe that the caregivers consider that the activities recommended by the system are appropriate (see Q2). However, it is observed that the conditions of the environment and the time in which the activities were done are not important. In particular, confirmed by Q3 where the percentage of undecideds is very high (40%). Finally, the caregiver positively perceives the patient’s performance of the activity, considering the activity to be adequate for the patient (see Q1). However, caregiver results should be taken very cautiously given the small number of responses, although the results obtained are quite promising.

## 6. Conclusions and Future Work

As has been shown, the use of technology to improve the lives of older people is a fundamental challenge in today’s society. With this in mind, this article proposes a cognitive assistant platform that aims to help people exercise. The platform, called ME^3^CA, calculates and adapts a set of personalised exercises to an elderly person at home.

The hardware consists of a series of biosensors, which are integrated into a chest belt and a bracelet. These sensors capture information that can be measured in the form of physical stress while the user performs the exercises and perceive its emotion. To interact with the users, ME^3^CA incorporates FitCLA, which suggests the most appropriate sequence of exercises according to the user’s profile and the values obtained from the biosensors, learning as the users evolve positively or negatively.

Our preliminary validation process of ME^3^CA has allowed us to reach some relevant conclusions. First, it is possible to attain the stress levels (in this case the effort level) of a person, as well as their emotional state and their vital signs, using cheap sensors with a high level of confidence. Also, learning procedures require a large amount of data to perform at an acceptable level of confidence (even if forced to overfit) and most unsupervised models achieve the same (low) accuracy values, thus simpler models can be used at this stage. Suggestions that are considered *common sense* are overlooked by the models, thus additional supervision is required by the caregiver. We believe that with more samples architectures, such as LSTM, could be more accurate as some of the fields that are used change rapidly over time, thus a more flexible model will perform better. Emotions introduce (currently) too much noise to be useful, which with more samples it could be reintroduced in the model. Although having environmental sensors serves to perform a preliminary filtering of the exercises to be suggested, it did not yield significant results on the learning models. We believe that it is due to the high variance of the sensor values. Finally, we observe that there is a shifting trend where care-receivers (elderly people) and caregivers are very inclined to use a technological platform to help them perform exercises more independently, even if it means that they have to “dress” themselves with several sensors. Although to confirm these findings a large study (in terms of time and people) is needed.

As future work, we aim to build a more complete dataset and with it test other machine learning methods for higher accuracy. Furthermore, we aim to produce miniature versions of the sensors and have them incorporated in specific clothing so that it is easier to use by the elders.

## Figures and Tables

**Table 1:** Hyperparameters of the network.

Layer (Type)	Output Shape	Param
$reshape_{1}$ (Reshape)	(None, 80, 6)	0
$conv1d_{1}$ (Conv1D)	(None, 71, 160)	9760
$conv1d_{2}$ (Conv1D)	(None, 62, 160)	256,160
$max_{p}ooling1d_{1}$ (MaxPooling1 (None, 20, 160)	–	0
$conv1d_{3}$ (Conv1D)	(None, 11, 160)	256,160
$conv1d_{4}$ (Conv1D)	(None, 2, 160)	256,160
$global_{a}verage_{p}ooling1d_{1}$ ( (None, 160)	–	0
$dropout_{1}$ (Dropout)	(None, 160)	0
$dense_{1}$ (Dense)	(None, 7)	1127

**Table 2:** Elderly questionnaire.

Exercise Identifier:	#Activity
Q1: I liked the activity	Y, N, No answer/don’t know
Q2: I felt good after the activity	Y, N, No answer/don’t know
Q3: I felt good before the activity	Y, N, No answer/don’t know
Q4: I have finished very excited	Y, N, No answer/don’t know
Q5: I have finished very bored	Y, N, No answer/don’t know
Q6: I have finished very overwhelmed	Y, N, No answer/don’t know
Q7: I have finished the activity with pain	Y, N, No answer/don’t know

**Table 3:** Caregiver Questionnaire.

Exercise & Patient Identifiers:	#Exercise, #Patient
Q1: The care receiver has done the suggested activity as it is described?	Very Low, Low, Normal, Well, Very Well
Q2: Suggested activity was appropriate for the patient	Very Low, Low, Normal, Well, Very Well
Q3: Suggested activity was appropriate at the time it was recommended	Very Low, Low, Normal, Well, Very Well
